# Nonlinear association between body mass index and psoriasis risk: A cross-sectional study based on the NHANES database

**DOI:** 10.1097/MD.0000000000049690

**Published:** 2026-07-10

**Authors:** Nuonan Lv, Minglin Zhang, Yusong Zhou, Yuxin Ming, Yulian Zhong, Zuojun Li

**Affiliations:** a Department of Pharmacy, The Third Xiangya Hospital, Central South University, Changsha, Hunan, China; b Department of Gastroenterology, The Third Xiangya Hospital, Central South University, Changsha, Hunan, China.

**Keywords:** body mass index, NHANES, obesity, psoriasis, threshold effect

## Abstract

Body mass index (BMI) is a widely used anthropometric measure reflecting overall adiposity. Emerging evidence suggests a significant association between obesity and psoriasis pathogenesis. This study aimed to investigate the nonlinear relationship between BMI and psoriasis risk, with particular focus on exploring BMI thresholds. Data were derived from the National Health and Nutrition Examination Survey (NHANES) database, spanning 2 nonconsecutive periods (2003–2006 and 2009–2014). The analytical sample comprised 19,892 participants aged ≥ 20 years who had complete information on BMI, psoriasis status, and all relevant covariates. The study utilized logistic regression to assess the association between psoriasis and BMI. The nonlinear relationship between BMI and psoriasis was examined using a generalized additive model (GAM) for smooth curve fitting. Additionally, a 2-piecewise linear regression model was fitted using a smoothing algorithm to identify potential critical BMI thresholds at which the association with psoriasis risk changed significantly. To explore potential effect modification, we conducted stratified analyses by several factors, including age, sex, and comorbidities. Curve fitting revealed a nonlinear positive association between BMI and psoriasis risk (*P* for nonlinearity < .001). A threshold effect was identified at BMI = 26.74 kg/m^2^: compared with BMI < 26.74 kg/m^2^, the odds ratio (OR) for psoriasis was 1.11 (95% CI: 1.05–1.16, *P* = .0002); for BMI ≥ 26.74 kg/m^2^, the magnitude of risk increase was attenuated (OR = 1.02, 95% CI: 1.00–1.03, *P* = .0394). Adjustments for covariates did not alter the robustness of these findings. The positive BMI-psoriasis association (per 5 kg/m^2^) remained consistent throughout subgroup analyses. In summary, we observed a significant association between higher BMI and increased psoriasis prevalence. However, the clinical significance of this association and its practical value in treatment management require further validation.

## 1. Introduction

Psoriasis is a chronic inflammatory skin disease affecting 2% to 3% of the global population, characterized by keratinocyte hyperproliferation and systemic immune dysregulation.^[1,2]^ Psoriasis is characterized not only by widespread recurrent skin plaques and patches but also frequently presents with multiple metabolic comorbidities, including obesity, hypertension, diabetes mellitus, dyslipidemia, nonalcoholic fatty liver disease (NAFLD), and metabolic syndrome (MetS).^[3-6]^

While genetic predisposition plays a pivotal role in psoriasis pathogenesis, emerging evidence indicates that obesity – a modifiable metabolic factor – serves as a significant contributor to the development and progression of psoriasis.^[7,8]^ Obesity is a chronic endocrine and metabolic disorder characterized by excessive adipose tissue accumulation and abnormal body weight gain.^[9,10]^ Accumulating evidence suggests that psoriasis and obesity share common underlying chronic inflammatory pathways, particularly involving pro-inflammatory cytokines such as TNF-α and IL-6.^[2,11,12]^

A prospective cohort study of US women demonstrated a significant linear positive association between body mass index (BMI) and psoriasis risk (*P* < .001).^[13]^ A prospective study conducted in female populations yielded consistent findings, demonstrating a graded increase in psoriasis risk with ascending BMI categories (*P* for trend < 0.001).^[14]^ Observational studies consistently report a positive association between elevated BMI (BMI ≥ 30 kg/m^2^) and psoriasis incidence (OR 1.5–2.0).^[15]^ Despite these advances, critical gaps remain in our understanding of the BMI-psoriasis relationship. Previous studies have predominantly assumed linear dose–response relationships, potentially failing to account for threshold effects where psoriasis risk exhibits either a disproportionate increase or decrease beyond specific BMI cut-points.^[16]^ Furthermore, the optimal BMI range for psoriasis prevention remains unexplored.

To investigate the association between BMI and psoriasis prevalence, we leverage the NHANES database to: examine the nonlinear relationship between BMI and psoriasis using GAM for smooth curve fitting, identify critical BMI thresholds using piecewise regression analysis, and validate findings using covariate-adjusted sensitivity analyses. These findings may provide potential information for future research on personalized weight management interventions for psoriasis prevention in at-risk populations.

## 2. Materials and methods

### 2.1. Data source, study population, and selection criteria

The study utilized data from the NHANES, an ongoing cross-sectional survey conducted by the National Center for Health Statistics (NCHS).^[17]^ The survey comprehensively assesses nutritional status, health behaviors, and clinical outcomes through interviews, physical examinations, and laboratory tests.^[18]^ The NHANES survey protocol was approved by the Institutional Review Board of the National Center for Health Statistics (NCHS), and all participants provided written informed consent. Detailed information on the study data can be accessed via the NHANES website (https://wwwn.cdc.gov/nchs/nhanes).

**Figure 1.**
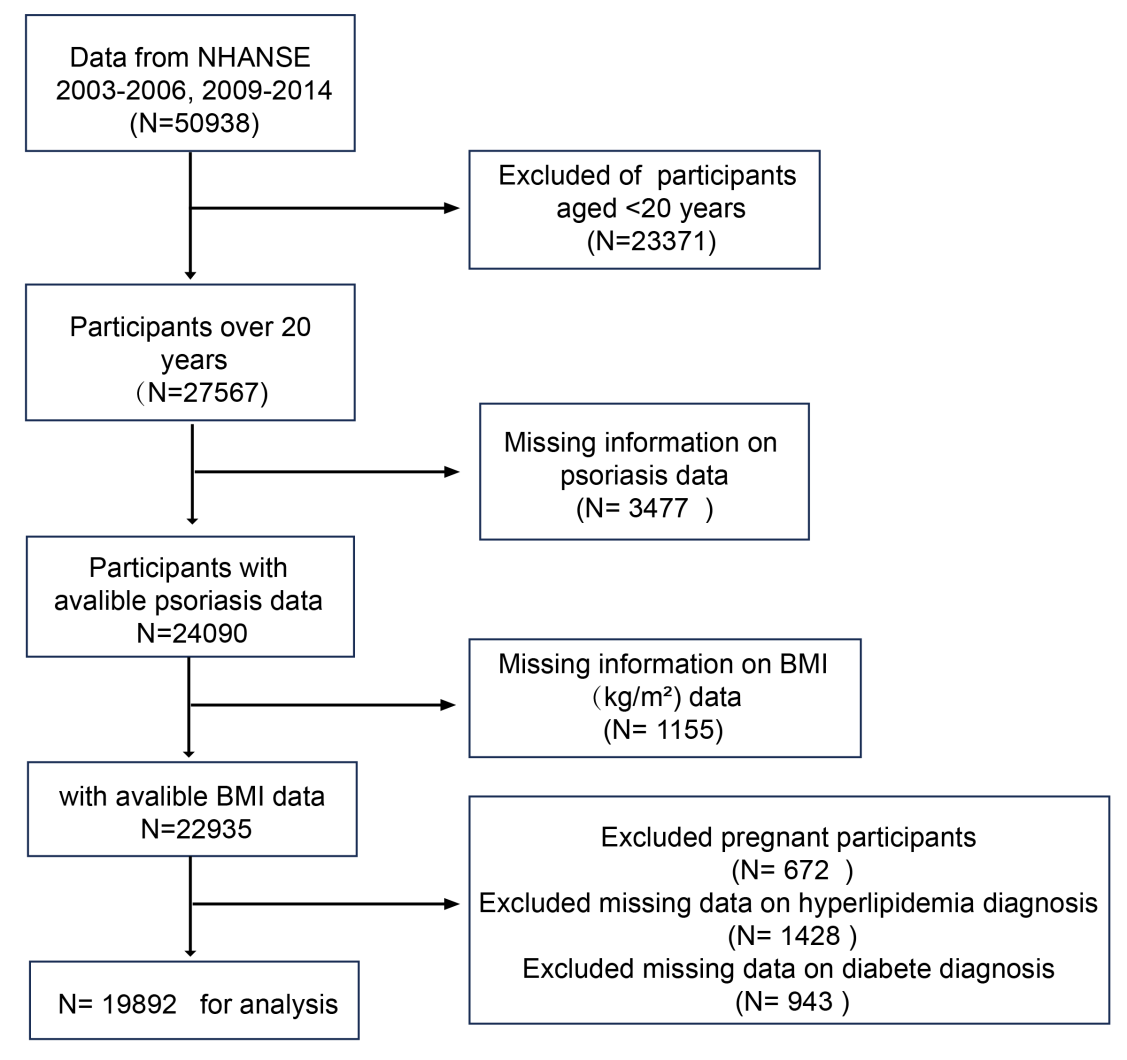
Participant selection flowchart. The study initially included 50,938 participants from NHANES survey cycles (2003–2006 and 2009–2014). A total of 23,371 individuals aged <20 years were excluded, retaining 27,567 adults. Subsequently, 3477 participants with missing psoriasis data were excluded, leaving 24,090 individuals with valid information. Following this, 1155 participants with missing BMI data were removed, resulting in 22,935 eligible individuals. Finally, the exclusion of 672 pregnant participants, 1428 individuals with missing hyperlipidemia data, and 943 individuals with missing diabetes diagnoses yielded a final analytical cohort of 19,892 participants. BMI = body mass index, NHANES = National Health and Nutrition Examination Survey.

The analysis utilized data from 50,938 participants across NHANES survey cycles (2003–2006, 2009–2014). The final analytical sample was selected according to the following criteria (Fig. 1): Inclusion criteria: aged 20 years or older and had complete data on psoriasis diagnosis status, body mass index (BMI), and all prespecified covariates (including hypertension, hyperlipidemia, and diabetes diagnoses). Exclusion criteria: Age < 20 years; missing data on psoriasis diagnosis; missing BMI data; pregnancy at the time of survey; missing data on hyperlipidemia diagnosis; missing data on diabetes diagnosis. The participant flow, including the sample size after each exclusion step, is detailed in Figure 1. After applying these criteria, a total of 19,892 participants were included in the final analysis.

### 2.2. Definition of psoriasis

Survey data from 5 collection cycles (2003–2006 and 2009–2014) of the NHANES included a specific inquiry regarding psoriasis diagnosis. Participants were asked, “Has a healthcare provider ever told you that you have psoriasis?” Those who responded affirmatively (“yes”) were categorized as having psoriasis for the purposes of this study, responses indicating uncertainty (“don’t know”), refusal to answer were excluded from the analysis to ensure the accuracy and reliability of the findings.^[8,19]^

### 2.3. Definition of BMI

In this study, we calculated the BMI using the standard formula: weight in kilograms divided by the square of height in meters. This calculation, expressed as kg/m^2^, was utilized to categorize the study population into 3 distinct groups based on BMI thresholds: individuals with a BMI <25 kg/m^2^ were classified as having normal weight, those with a BMI between 25 and 30 kg/m^2^ were considered overweight, and those with a BMI >30 kg/m^2^ were identified as obese.^[20]^

### 2.4. Covariates

Based on reviews of the literature, we rigorously selected a set of covariates to mitigate potential confounding biases. These variables encompassed demographic characteristics (gender, age, and race/ethnicity – specifically non-Hispanic White, non-Hispanic Black, Mexican American, other Hispanic, and other racial/ethnic groups), socioeconomic indicators (educational attainment categorized as less than high school, and high school or above; and household income relative to the poverty threshold, stratified into >3.5, >1.3, ≤3.5, and ≤1.3), and lifestyle-related factors (alcohol intake, defined as ≥ 12 alcoholic drinks annually; and cigarette smoking, defined as lifetime consumption exceeding 100 cigarettes). Hypertension diagnosis was based on self-report or blood pressure measurements (systolic ≥ 140 mm Hg and/or diastolic ≥ 90 mm Hg). Diabetes was identified using the following criteria: self-reported diagnosis via questionnaires; utilization of insulin or diabetes-related medications; fasting glucose levels of at least 126 mg/dL; and glycated hemoglobin A1c (HbA1c) levels of 6.5% or higher. Hyperlipidemia was characterized by total cholesterol levels of 240 mg/dL or higher, LDL cholesterol levels of 130 mg/dL or higher, triglyceride levels of 150 mg/dL or higher, HDL cholesterol levels below 40 mg/dL in men or below 50 mg/dL in women, or by the self-reported use of lipid-lowering medications. The selection of these covariates was guided by prior epidemiological evidence, clinical relevance, and statistical assessments to ensure robust control of confounding.

### 2.5. Statistical analysis

Statistical analyses were conducted in strict accordance with the methodological guidelines established by the US Centers for Disease Control and Prevention (CDC). We implemented a standardized protocol for missing data: variables with over 10% missing values were omitted. For categorical variables, missing values were imputed using the mode. For continuous variables, the imputation strategy was based on their distribution: the mean was applied to normally distributed data, whereas the median was used for data with a non-normal distribution. Statistical significance was determined using 2-sided tests with a threshold of *P* < .05. All analyses were performed in R (version 4.4.2) and Empower Stats (version 5.2).

**Figure 2.**
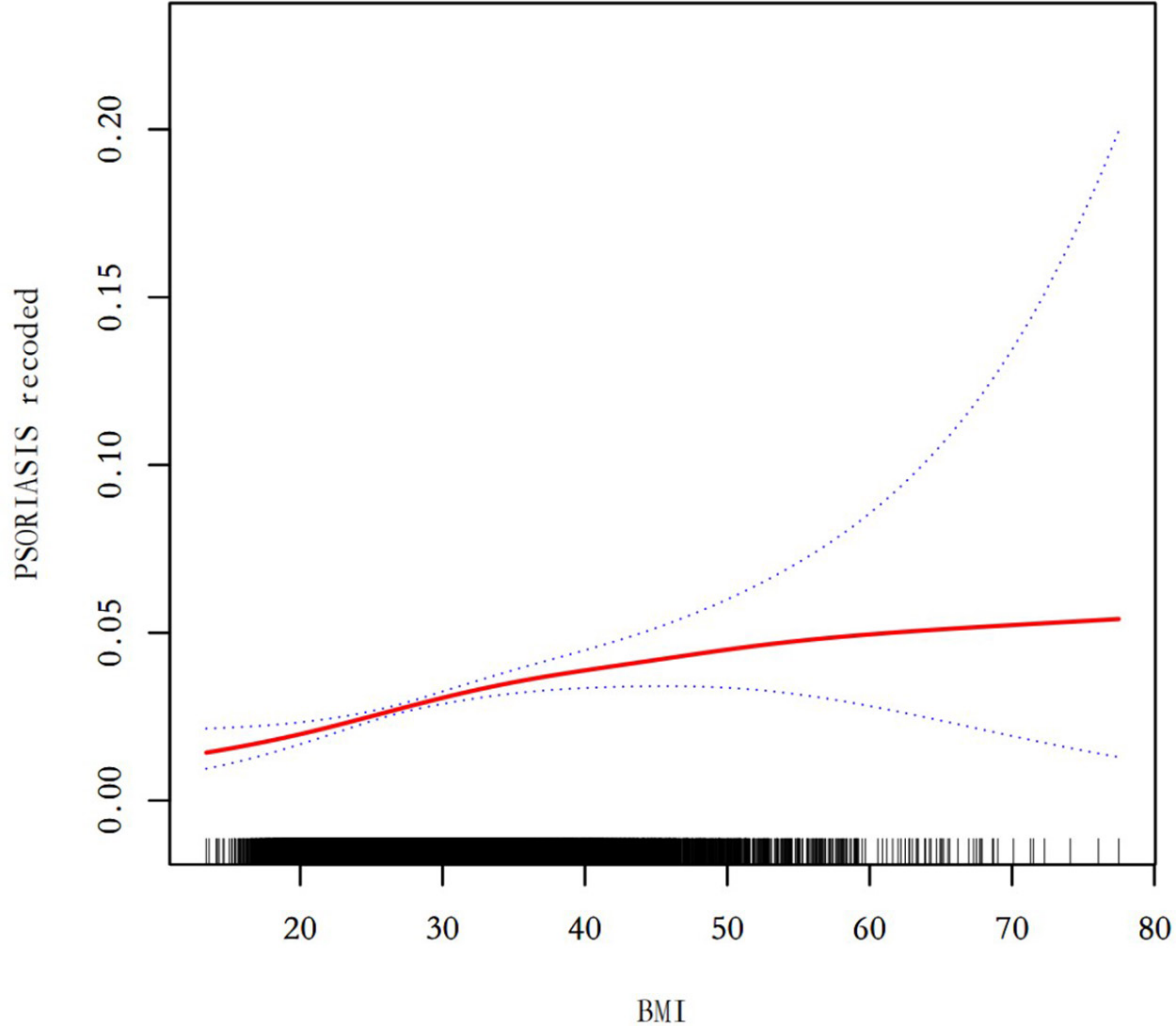
Nonlinear association between BMI and psoriasis probability. Nonlinear positive association between BMI and psoriasis (*P* for nonlinearity < .001). The solid red line represents the smooth curve fit between variables. The area between 2 blue dashed lines indicates the fitted 95% confidence interval. BMI = body mass index.

Descriptive statistical analyses were conducted, with continuous variables quantified as mean ± standard deviation (SD) and categorical variables reported as frequency counts (%). Differences in baseline covariates between psoriasis patients and control subjects were assessed using independent samples *t* tests (for continuous metrics) and chi-squared tests (for categorical classifications; Table 1). A GAM was employed to analyze the nonlinear association between BMI and psoriasis risk (Fig. 2). Through threshold effect modeling, we determined clinically significant BMI cut-points associated with altered psoriasis risk profiles. We assessed the existence of a threshold effect by comparing a simple linear model (model I) against a 2-segment piecewise linear regression model (model II) that incorporated a single threshold. The superior fit of the piecewise model was formally tested using the log-likelihood ratio (LLR) test (Table 2).

**Table 1:** Characteristics of participants (N = 19,892).

Characteristics	Total (N = 19,892)	Non-psoriasis (n = 19325)	Psoriasis (n = 567)	*P*-value
Age (yr)	47.64 ± 16.69	47.56 ± 16.71	50.35 ± 15.87	<.001
Sex, %				.699
Male	9772 (49.13)	9498 (49.15)	274 (48.32)	
Female	10,120 (50.87)	9827 (50.85)	293 (51.68)	
Race/ethnicity, %				<.001
Mexican American	3059 (15.38)	3014 (15.60)	45 (7.94)	
Other Hispanic	1682 (8.46)	1637 (8.47)	45 (7.94)	
Non-Hispanic White	8874 (44.61)	8533 (44.16)	341 (60.14)	
Non-Hispanic Black	4231 (21.27)	4155 (21.50)	76 (13.40)	
Othe race	2046 (10.29)	1986 (10.28)	60 (10.58)	
Education level, %				.015
Below high school	4751 (23.88)	4640 (24.01)	111 (19.58)	
High school or above	15,141 (76.12)	14,685 (75.99)	456 (80.42)	
Marital status, %				.868
Cohabitation	18,558 (93.29)	18,030 (93.30)	528 (93.12)	
Solitude	1334 (6.71)	1295 (6.70)	39 (6.88)	
PIR, %				.042
≤1.3	5933 (29.83)	5750 (29.75)	183 (32.28)	
>1.3, ≤3.5	7997 (40.20)	7798 (40.35)	199 (35.10)	
>3.5	5962 (29.97)	5777 (29.89)	185 (32.63)	
Smoking status, %				<.001
Yes	8926 (44.87)	8609 (44.55)	317 (55.91)	
No	10,966 (55.13)	10,716 (55.45)	250 (44.09)	
Drinking status, %				.411
Yes	13,165 (73.10)	12,773 (73.05)	392 (74.67)	
No	4845 (26.90)	4712 (26.95)	133 (25.33)	
Diabetes, %				.015
Yes	3226 (16.22)	3113 (16.11)	113 (19.93)	
No	16,666 (83.78)	16,212 (83.89)	454 (80.07)	
Hypertension, %				.168
Yes	3263 (16.40)	3158 (16.34)	105 (18.52)	
No	16,629 (83.60)	16,167 (83.66)	462 (81.48)	
Hyperlipidemia, %				.005
Yes	4141 (20.82)	4050 (20.96)	91 (16.05)	
No	15,751 (79.18)	15,275 (79.04)	476 (83.95)	
BMI, kg/m^2^, %				<.001
≤25	5742 (28.87)	5621 (29.09)	121 (21.34)	
>25, ≤30	6612 (33.24)	6415 (33.20)	197 (34.74)	
>30	7538 (37.89)	7289 (37.72)	249 (43.92)	

BMI = body mass index, PIR = poverty-to-income ratio, *P*-value = probability value.

**Table 2:** Threshold effect analysis of BMI and psoriasis using piece-wise linear regression.

Model	OR (95% CI)	*P*-value
Model 1, linear effect model	1.03 (1.02, 1.04)	<.0001
Model 2, turning point (K)	26.74	
Odds ratio < K	1.11 (1.05, 1.18)	.0003
Odds ratio ≥ K	1.02 (1.00, 1.03)	.0414
LLR	.006	
95% CI of K	26.74 (26.00, 28.05)	

Threshold effect analysis of BMI and psoriasis. Data were presented as OR (95% CI), *P* value; model I, linear analysis; model II, nonlinear analysis. Adjusted for age (years), sex, race, education-level, PIR, smoking-status, drinking-status.

BMI = body mass index, CI = confidence interval, OR = odds ratio, LLR = log-likelihood ratio test, PIR = poverty-to-income ratio, *P*-value = probability value.

**Figure 3.**
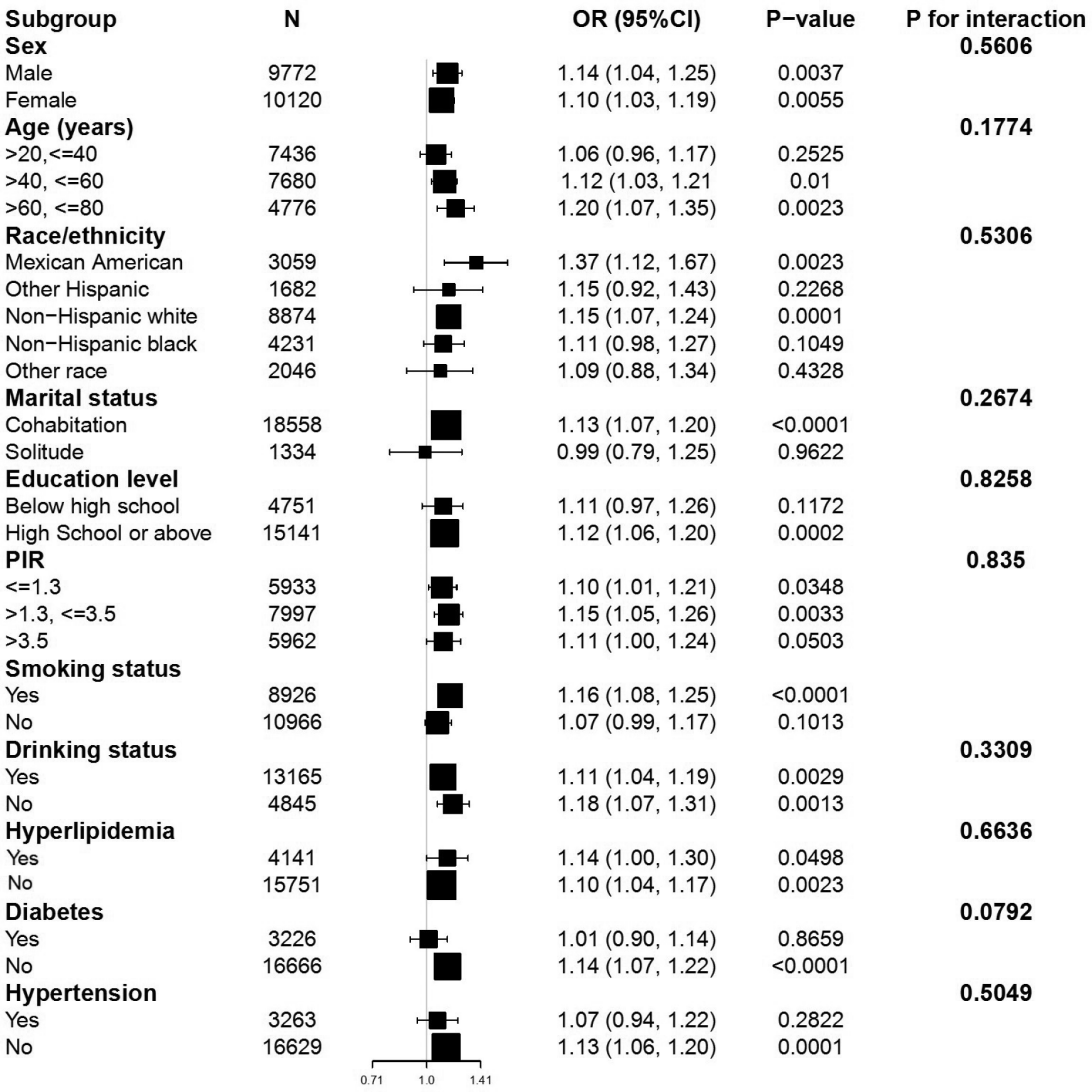
The association between BMI and psoriasis by different subgroups, per 5 units. BMI = body mass index, CI = confidence interval, OR = odds ratio, PIR = poverty-to-income ratio, *P*-value = probability value.

Logistic regression models were used to investigate the relationship between the BMI index and psoriasis. Covariates were not adjusted in the crude model (model 1). The minimally adjusted model (model 2) was adjusted for age, sex, race, and education level. The fully adjusted model (model 3) was adjusted for age, sex, race, education level, marital status, PIR, smoking, alcohol intake, hypertension, and diabetes (Table 3). To evaluate the robustness of the association between BMI and psoriasis, subgroup analyses were performed across various population characteristics (Fig. 3).

**Table 3:** Association between BMI and psoriasis in NHANES 2003–2006 and 2009–2014.

Exposure	Non-adjusted	Model 2	Model 3
BMI	1.0 (1.0, 1.0) < 0.001	1.0 (1.0, 1.0) < 0.001	1.0 (1.0, 1.0) < 0.001
BMI categorical			
≤25	1.0	1.0	1.0
>25, ≤30	1.4 (1.1, 1.8) 0.002	1.5 (1.2, 1.9) < 0.001	1.5 (1.2, 1.9) 0.002
>30	1.6 (1.3, 2.0) < 0.001	1.8 (1.4, 2.2) < 0.001	1.7 (1.4, 2.2) < 0.001
*P* for trend	<.001	<.001	<.001

Statistical analysis: Logistic regression models were used to calculate OR and 95% CI. BMI was analyzed as both a continuous variable and categorical groups (≤25, >25, ≤30, >30), with psoriasis as the dependent variable. Non-adjusted (model 1) adjusted for: None. Model 2 adjusted for: age (years), sex, race. Model 3 adjusted for: age (years), sex, race, education level, PIR, smoking history, marital status, drinking status, lipids, hypertension, hyperlipidemia, diabetes.

BMI = body mass index, CI = confidence interval, NHANES = National Health and Nutrition Examination Survey, OR = odds ratio, PIR= poverty-to-income ratio *P*-value = probability value.

## 3. Results

### 3.1. Baseline characteristics

The study included 19,892 participants (2.85% with psoriasis). Psoriasis patients were significantly older (50.35 vs 47.56 years, *P* < .001), more likely to be non-Hispanic White (60.14% vs 44.16%, *P* < .001), and had higher rates of obesity (43.92% vs 37.72%, *P* < .001), smoking (55.91% vs 44.55%, *P* < .001), and diabetes (19.93% vs 16.11%, *P* = .015). Socioeconomic analysis revealed psoriasis patients were more likely to have lower income (PIR ≤ 1.3: 32.28% vs 29.75%, *P* = .042). No significant differences were observed for sex, marital status, drinking status, or hypertension (Table 1).

### 3.2. Nonlinear association between BMI and psoriasis probability

Figure 2 illustrates the nonlinear positive relationship between BMI and the risk of psoriasis. The solid red line represents the odds ratio (OR) of psoriasis associated with different levels of BMI, while the blue dashed lines denote the 95% confidence intervals (CI). The analysis reveals a significant nonlinear association between BMI and psoriasis risk (*P* < .001).

### 3.3. Threshold effect analysis

Threshold effect analysis confirmed significant nonlinearity (likelihood ratio test *P* = .006). The linear model (model I) showed a marginal but statistically significant association (OR = 1.03 per 1-unit BMI increase, 95% CI: 1.02–1.04, *P* < .0001). However, piecewise regression (model II) identified a clinically relevant threshold at BMI = 26.74 kg/m^2^ (95% CI: 26.0–28.05), with distinct risk patterns: below this cutoff, each BMI unit increased psoriasis risk by 11% (OR = 1.11, 95% CI: 1.05–1.18, *P* = .0003), whereas above it, the effect attenuated to 2% (OR = 1.02, 95% CI: 1.00–1.03, *P* = .0414; Table 2).

### 3.4. The association between psoriasis and BMI

Table 3 demonstrates a significant positive association between BMI and psoriasis prevalence across all models (*P*-trend < .001). In the fully adjusted model (model 3), overweight (BMI 25–30 kg/m^2^) and obese (BMI > 30 kg/m^2^) participants had 50% (OR = 1.5, 95% CI: 1.2–1.9, *P* = .002) and 70% (OR = 1.7, 95% CI: 1.4–2.2, *P* < .001) higher odds of psoriasis, respectively, compared to normal-weight individuals (BMI ≤ 25 kg/m^2^). Consistent dose–response relationships (*P*-trend < .001) and robustness across different adjusted models indicate a strong association between psoriasis and BMI.

### 3.5. Subgroup analysis and interactions

Subgroup analyses demonstrated a consistent positive association between BMI (per 5-unit increment) and psoriasis (Fig. 3). The association was particularly pronounced in older adults (>60 years), Mexican Americans, and smokers, while no significant effect modification was observed by sex, marital status, or socioeconomic factors.

## 4. Discussion

Our study demonstrates a nonlinear positive association between BMI and psoriasis risk, with a critical threshold identified at 26.74 kg/m^2^. Below this threshold, each 1-unit increase in BMI was associated with an 11% higher risk of psoriasis (OR = 1.11, 95% CI: 1.05–1.18, *P* = .0003), while above the threshold, the risk plateaued (OR = 1.02, 95% CI: 1.00–1.03, *P* = .0414), suggesting a departure from the conventional linear assumption and underscoring the importance of targeted weight management strategies, particularly for individuals with a BMI approaching or exceeding this critical threshold.

One study reported a 9% increase in psoriasis risk per 1 kg/m^2^ increment in BMI (OR = 1.09, 95% CI: 1.06–1.12),^[15]^ while another employing genetic correlation and Mendelian randomization methods further corroborated this association, suggesting that obesity may play a role in psoriasis development.^[21]^ Complementing these findings, a prospective meta-analysis identified a nonlinear relationship, with the lowest risk observed at approximately 20 kg/m^2^, a gradual increase beyond this threshold, and accelerated progression at BMI levels (≥25 kg/m^2^).^[16]^ Using NHANES data, 1 study demonstrated that abdominal obesity indicators – including the conicity index, android fat percentage, and body roundness index – are positively associated with psoriasis risk.^[22]^ Similarly, relative fat mass (RFM), a novel estimate of total body fat, has been investigated for its role in psoriasis. One analysis found that each unit increase in RFM was associated with 7% higher odds of psoriasis, with RFM demonstrating superior predictive capacity compared to traditional indices such as BMI, weight-adjusted waist index, and body roundness index.^[23]^ However, another cross-sectional study reported a more modest association (OR = 1.03 per unit increase) and noted that the predictive ability of RFM was limited (AUC = 0.549), with the poverty income ratio acting as a significant effect modifier.^[24]^ Collectively, these findings underscore the complexity of the relationship between body composition and psoriasis risk, suggesting that both the choice of adiposity measure and consideration of sociodemographic factors may influence observed effect estimates.

Biological plausibility and mechanistic insights. Psoriasis, as a chronic inflammatory disorder, may exhibit a characteristic dose–response relationship with adiposity: Mild BMI elevation could initiate disease by activating susceptibility genes, while severe obesity may show attenuated risk progression due to metabolic compensation mechanisms.^[25,26]^ The observed threshold effect aligns with established biological mechanisms linking adiposity to psoriatic inflammation. Accumulating evidence has established the IL-23/Th17 axis as the central immunological pathway in psoriasis pathogenesis, driving both inflammatory cascades and keratinocyte dysregulation.^[1,20,27]^ Excess adipose tissue in obesity secretes elevated levels of pro-inflammatory adipokines, which directly enhance Th17 cell differentiation and IL-23 receptor expression.^[28,29]^ This endocrine dysfunction potentiates psoriatic inflammation through sustained activation of the IL-23/Th17 axis, ultimately accelerating disease progression.^[28,30]^ This bidirectional relationship between psoriasis and obesity creates a self-perpetuating cycle, complicating clinical management and highlighting the need for integrated therapeutic strategies.^[9,16,31]^

Obesity has been shown to compromise treatment efficacy across various psoriasis therapies. Excess adipose tissue may reduce cutaneous drug penetration of topical agents, while altered pharmacokinetics in systemic therapies often necessitate dose adjustments for optimal therapeutic outcomes.^[32,33]^ Previous clinical studies have demonstrated that dietary interventions achieving 10% to 15% weight loss resulted in significant improvement in psoriasis severity among obese patients, with a mean 48% reduction in Psoriasis Area and Severity Index (PASI) scores (*P* < .001).^[34]^ Collectively, weight management emerges as a potential preventive strategy against psoriasis development.^[35-37]^ Future research should clarify the biological mechanisms linking BMI to psoriasis pathogenesis, while concurrently investigating BMI-based therapeutic stratification in clinical settings.

This study has several notable strengths. By leveraging a large US population-based dataset, we ensured a robust sample size, which strengthened the statistical power. Additionally, our exploration of obesity as a critical factor in the relationship between psoriasis and BMI offers valuable new insights and opportunities for future research. However, some limitations should be considered. First, cross-sectional studies provide a snapshot of associations at a single time point but cannot establish causality. Second, self-reported psoriasis diagnoses, as used in this study, carry the risk of misclassification bias, as participants might confuse psoriasis with other skin conditions. Third, since psoriasis severity was not assessed in this study, we were unable to examine potential associations between disease severity and degree of adiposity. Finally, the inclusion/exclusion criteria may have introduced selection bias, potentially overrepresenting participants with complete data while underrepresenting those with missing values, who might constitute a clinically distinct subgroup with higher comorbidity burden. Given potential differences in genetic, lifestyle, and environmental factors across diverse populations, further studies in various ethnic and regional groups are necessary to validate and understand the universality of the relationship between BMI changes and psoriasis risk.

## 5. Conclusion

In conclusion, our study identifies a nonlinear association between BMI and psoriasis risk. Additionally, several key risk factors were identified: non-Hispanic White ethnicity, smoking history, and diabetes mellitus. These findings provide epidemiological evidence for the prevention and management of psoriasis, suggesting that weight control and lifestyle modifications may serve as effective strategies to reduce psoriasis incidence. Further studies are needed to elucidate the biological mechanisms underlying the BMI-psoriasis association and explore actionable interventions to optimize BMI control for psoriasis prevention or treatment.

## Acknowledgments

We thank the NHANES database team for providing access to the data.

## Author contributions

**Data curation:** Yusong Zhou.

**Formal analysis:** Nuonan Lv, Minglin Zhang.

**Investigation:** Yusong Zhou, Yuxin Ming.

**Methodology:** Nuonan Lv, Yulian Zhong.

**Software:** Nuonan Lv, Minglin Zhang.

**Supervision:** Minglin Zhang.

**Writing – original draft:** Nuonan Lv.

**Writing – review & editing:** Zuojun Li.
